# Prognostic Significance of Signet-Ring Cell Components in Patients With Gastric Carcinoma of Different Stages

**DOI:** 10.3389/fsurg.2021.642468

**Published:** 2021-07-15

**Authors:** Xiaoyuan Dong, Guorui Sun, Hui Qu, Qingsi He, Zhaofan Hao

**Affiliations:** ^1^Department of Hematology, Qilu Hospital of Shandong University, Jinan, China; ^2^Department of General Surgery, Qilu Hospital of Shandong University, Jinan, China; ^3^Department of Nephrology, Eastern District, Qilu Hospital of Shandong University, Jinan, China

**Keywords:** gastric cancer, signet-ring cell adenocarcinoma, surgery, prognosis, treatment

## Abstract

**Background:** Gastric carcinoma (GC), which contains signet ring cell (SRC) components are frequently observed in postoperative pathological assessment. This study aims to study the prognostic significance of SRC components in GC patients.

**Methods:** From 2003 to 2017, surgically resected primary GC patients were retrospectively reviewed. All enrolled patients were divided into three groups according to the proportion of SRC. The overall survival (OS) and disease-free survival (DFS) of GC patients with different tumor stages were analyzed.

**Results:** Patients with SRC or mixed-SRC were more associated with female, younger age, middle or lower third of the stomach, larger tumor, higher pN stage, and more lymphovascular invasion. For GC patients in stage I, multivariate survival analysis showed that age >60, SRC components >50%, and pT stage were independent prognostic factors for OS (all *p* < 0.05). The 5-year OS of patients with SRC were higher than that of patients with pure adenocarcinoma (*p* = 0.021). For GC patients in stage II/III, multivariate survival analysis showed that age >60, SRC proportion, surgical types, Borrmann's type, pT stage, pN stage, and lymphovascular invasion were independent prognostic factors for OS (all *p* < 0.05). The 5-year OS/DFS of patients with SRC were lower than that of patients with pure adenocarcinoma (*p* < 0.001).

**Conclusions:** SRC seemed to be a favorable prognostic factor in GC patients in stage I. However, for GC patients in stage II/III, the SRC components were associated with poor prognosis, independent of other clinicopathological factors.

## Introduction

Gastric carcinoma (GC) contains a group of histopathological heterogeneous components, such as adenocarcinoma, signet ring cell (SRC) carcinoma, neuroendocrine carcinoma, squamous cell carcinoma, etc. In GC, adenocarcinoma is the most common pathological type, which accounts for about 90% of all GC cases (1). The SRC carcinoma only account for only about 5–10% of all GC cases (2). The malignant pathological features of SRC, including more lymph node metastasis, easier distant metastasis, and late staging have been widely recognized (3). The 2010 World Health Organization (WHO) pathological classification defines SRC based on the proportion of the main components (>50%) (4). However, gastric adenocarcinoma mixed with SRC components (mixed-SRC) are frequently observed in the clinic, which refers to a mixture of adenocarcinoma and SRC components of 50% or less. However, clinicopathological characteristics and prognosis of SRC components are yet to be fully clarified (5).

Some studies have revealed the different prognostic significance of SRC in early or advanced GC patients. In general, SRC implies worse prognosis in patients with advanced GC (6). Interestingly, for early GC patients, SRC often means favorable prognosis than common adenocarcinomas (7). It is still unclear whether GC patients with mixed-SRC follow the same principles. This study aims to investigate the clinicopathological characteristics and prognostic significance of SRC components in patients with GC of different stages.

## Materials and Methods

### Patients

**Figure 1:**
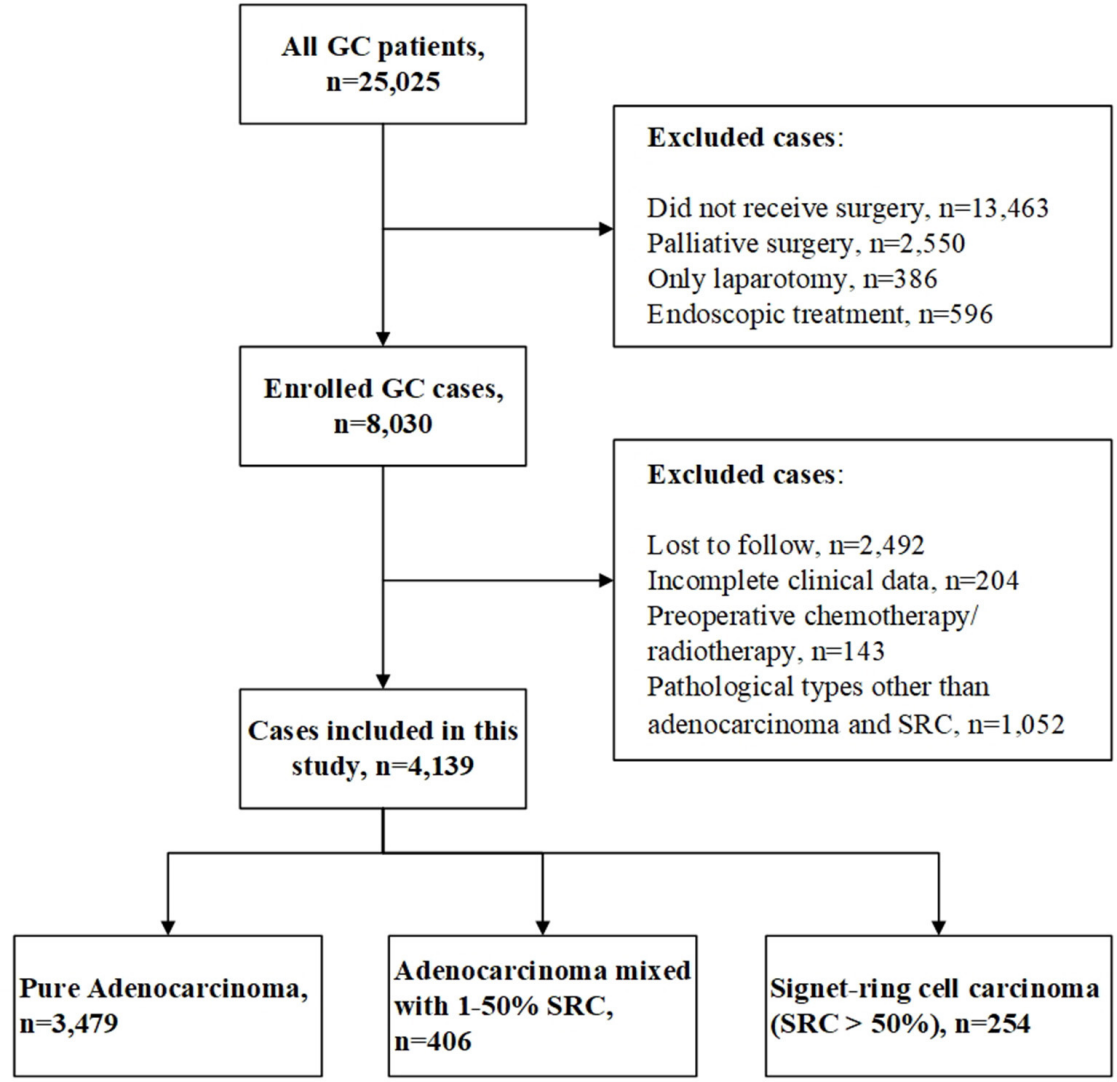
Flowchart of patients' selection process (GC, gastric cancer; SRC, signet ring cell).

We retrospectively analyzed 21,327 GC cases in the Qilu Hospital of Shandong University from January 2003 to December 2017. The inclusion criteria were as follows: (1) pathological diagnosis as primary GC and (2) patients underwent radical gastrectomy with D2 lymphadenectomy. The exclusion criteria were as follows: (1) patients received neoadjuvant chemotherapy or radiotherapy before surgery, (2) patients had multiple gastric primary tumors, and (3) adenocarcinoma with other pathological types of differentiated tissues except for SRC, such as mucinous adenocarcinoma, neuroendocrine differentiation, squamous cell carcinoma, etc. In our study, most patients in stage II/III received adjuvant chemotherapy after surgery. Patients who did not received adjuvant chemotherapy were those of old age, poor physical fitness, taboo cardiopulmonary function, or refusal of treatment. The chemotherapy regimens we performed on these patients included SOX (S-1 + oxaliplatin), XELOX (capecitabine + oxaliplatin), and FOLFOX (5-Fu + tetrahydrofolate + oxaliplatin). All patients were followed-up by telephone or outpatient after surgery. The following-up information included the date of follow-up, date of tumor recurrence/metastasis, and the date and cause of death. The final follow-up was December 2019. The median follow-up period was 84.0 months (range, 20.0–190.0 months). This study was approved by the Ethics Committee of Qilu Hospital of Shandong University [No. KYLL-2019(KS)-487]. The patients' selection processing is shown in Figure 1.

### Histopathological Evaluation

We retrospectively reviewed pathology reports of all included cases. The following data were collected: age, gender, tumor location, pathological diagnosis, SRC differentiation proportion, pathological tumor stage, surgical type, lymphovascular invasion, and perineural invasion. For the pathology reports before the year of 2010, which did not indicate exact proportion of SRC components (*n* = 504, 12.2%), pathological slides were retrieved and diagnosed by two experienced independent pathologists (C.S.M. and L.L.) (8). Pathologic tumor staging was defined by the American Joint Committee on Cancer (AJCC) eighth edition tumor–node–metastasis (TNM) staging system. An SRC signifies that cells secrete a large amount of mucus in the cytoplasm and squeezes the nucleus to one side, and the nucleus is crescent shaped (9). The cutoff value of 50% was based on the 2010 WHO Classification of Tumors of the Digestive System, which defined SRC as a carcinoma with more than 50% of SRC components. Adenocarcinomas containing 1–50% SRC components were defined as mixed-SRC (4).

### Statistical Analysis

The Chi-square test and *t*-test are used in the comparison of different subgroups for clinicopathological characteristics. The Kaplan–Meier method was used to calculate the OS curves based on the length of time between primary surgical treatment and final follow-up or death, and DFS curves based on the length of time between primary surgical treatment and final follow-up or death or recurrence or metastasis. The log-rank test was used to assess statistical differences between curves. Univariate and multivariate Cox proportional hazards regression model were used to identify independent prognostic factors. A *p* < 0.05 was considered statistically significant. The statistical analysis was performed using SAS 9.4 (SAS Institute Inc., Cary, NC, USA).

## Results

### Comparison of Clinicopathological Characteristics in Three Subgroups

A total of 4,139 cases were enrolled in this study, including 1,640 laparoscopic gastrectomy. According to the proportion of SRC components (0, 1–50, or >50%), all cases were categorized into three groups: (1) 3,479 pure adenocarcinoma cases (without SRC component), (2) 406 adenocarcinomas cases mixed with SRC (SRC component 1–50%), and (3) 254 SRC cases (SRC component >50%). Patients with mixed-SRC were more associated with female, younger age, middle or lower third of the stomach, total gastrectomy, higher pN stage, Borrmann's type II, and more lymphovascular invasion (all *p* < 0.05). Patients with SRC were more associated with female, younger age, middle or lower third of the stomach, larger tumor, higher pN stage, and Borrmann's type III/ IV (all *p* < 0.05). The 5-year OS rate of pure adenocarcinoma, mixed-SRC, and SRC were 73.16, 69.32, and 65.82%, respectively (*p* = 0.013). The 5-year DFS rate of pure adenocarcinoma, mixed-SRC, and SRC were 75.65, 73.13, and 66.63%, respectively (*p* = 0.048). Detailed data are listed in Table 1 (*p* < 0.05 are in bold print).

**Table 1:** Clinicopathological characteristics of different groups according to the SRC proportion.

**Variables**	**Pure AC** **(without SRC)** ***n* = 3,479**	**Mixed-SRC** **(1–50% SRC)** ***n* = 406**	**SRC (SRC > 50%)** ***n* = 254**	***t*/χ^2^**	***p*-value**
Gender^*^^#^				59.33	**<0.001**
Male	2,769 (79.59)	277 (68.23)	160 (62.99)		
Female	710 (20.41)	129 (31.77)	94 (37.01)		
Age (years)^*^^#^				47.89	**<0.001**
≤ 60	1,523 (43.78)	244 (60.1)	140 (55.12)		
>60	1956 (56.22)	162 (39.9)	114 (44.88)		
Tumor location^*^^#^				150.07	**<0.001**
Upper third	1,559 (44.81)	93 (22.91)	58 (22.83)		
Middle third	619 (17.79)	127 (31.28)	72 (28.35)		
Lower third	1,259 (36.19)	167 (41.13)	113 (44.49)		
Whole stomach	44 (1.27)	18 (4.36)	11 (4.33)		
Tumor diameter (mean ± SD, cm)^#^	4.47 ± 2.57	4.70 ± 3.17	5.01 ± 3.17	5.84	**0.003**
Surgical type^*^				17.58	**<0.001**
Subtotal	2,799 (80.45)	297 (73.15)	191 (75.2)		
Total	673 (19.34)	106 (26.11)	62 (24.41)		
Combined organs	7 (0.2)	3 (0.74)	1 (0.39)		
pT stage				9.88	0.130
T1	653 (18.77)	90 (22.17)	62 (24.41)		
T2	437 (12.56)	50 (12.32)	21 (8.27)		
T3	707 (20.32)	78 (19.21)	48 (18.9)		
T4	1,682 (48.35)	188 (46.31)	123 (48.43)		
No. of lymph node dissected [median (range)^§^]	19 (14–25)	22 (17–29)	20 (15–27)	7.23	0.115
pN stage^*^^#^				34.66	**<0.001**
N0	1,410 (40.53)	147 (36.21)	101 (39.76)		
N1	606 (17.42)	44 (10.84)	33 (12.99)		
N2	634 (18.22)	79 (19.46)	37 (14.57)		
N3	829 (23.83)	136 (33.5)	83 (32.68)		
pTNM				7.15	0.130
I	860 (24.72)	107 (26.35)	69 (27.17)		
II	896 (25.75)	87 (21.43)	51 (20.08)		
III	1,723 (49.53)	212 (52.22)	134 (52.76)		
Borrmann's type^*^^#^				154.61	**<0.001**
I	378 (13.37)	21 (6.65)	11 (5.73)		
II	367 (12.98)	113 (35.76)	42 (21.88)		
III	1,692 (59.83)	127 (40.19)	92 (47.92)		
IV	391 (13.83)	55 (17.41)	47 (24.48)		
Lymphovascular invasion^*^				10.43	**0.010**
Yes	508 (14.6)	84 (20.69)	39 (15.35)		
No	2,971 (85.4)	322 (79.31)	215 (84.65)		
Perineural invasion				1.88	0.390
Yes	142 (4.08)	20 (4.93)	7 (2.76)		
No	3,337 (95.92)	386 (95.07)	247 (97.24)		
Adjuvant chemotherapy				5.46	0.070
Yes	1,554 (63.17)	222 (69.81)	113 (64.94)		
No	906 (36.83)	96 (30.19)	61 (35.06)		
5-year OS rate (%)	73.16	69.32	65.82	8.64	**0.013**
5-year DFS rate (%)	75.65	73.13	66.63	6.09	**0.048**

*AC, adenocarcinoma; SRC, signet ring cell; pT, pathological tumor; pN, pathological node; pTNM, pathological tumor–node–metastasis; OS, survival; DFS, disease-free survival*.

* *Represents statistically significant differences between pure AC and mixed-SRC groups*.

# *Represents statistically significant differences between pure AC and SRC groups*.

§ *Interquartile range*.

*All p <0.05 are marked in bold print*.

### Univariate and Multivariate Survival Analysis of Overall Survival in Patients With Stage I Gastric Cancer

We subsequently evaluated clinicopathological factors associated with OS in GC patients in stage I. In univariate analysis, age >60 (*p* = 0.001), SRC components >50% (*p* = 0.047), and pT stage (*p* = 0.000) were prognostic factors for OS (Table 2). However, parameters such as gender, tumor location, surgical type, pN stage, lymphovascular invasion, and perineural invasion were not prognostic factors for OS (all *p* > 0.05, Table 2). In multivariate survival analysis, age >60 (*p* = 0.002), SRC components >50% (*p* = 0.040), and pT stage (*p* < 0.001) were independent prognostic factors for OS (Table 2).

**Table 2:** Univariate and multivariate COX regression analysis for OS of patients with stage I GC.

**Variables**	**No. of patients** **(*n* = 1,036)**	**Univariate analysis**			**Multivariate analysis**		
		**Hazard ratio** **(95% CI)**	**χ^2^**	***p-*value**	**Hazard ratio** **(95% CI)**	**χ^2^**	***p*-value**
**Gender**							
**Male**	790 (76.25)	0.89 (0.55–1.42)	0.24	0.624			
**Female**	246 (23.75)	1.00					
**Age (years)**							
**≤60**	511 (49.32)	1.00			1.00		
**>60**	525 (50.68)	1.95 (1.30–2.94)	10.27	**0.001**	1.91 (1.26–2.89)	9.23	**0.002**
**Tumor location**							
**Upper third**	219 (21.14)	1.00					
**Middle third**	217 (20.95)	0.83 (0.46–1.48)	0.41	0.521			
**Lower third**	600 (57.92)	0.82(0.51–1.30)	0.72	0.397			
**SRC proportion**							
**0% (pure AC)**	860 (83.01)	1.00			1.00		
**1–50% (mixed-SRC)**	107 (10.33)	1.26 (0.69–2.30)	0.56	0.455	1.27 (0.70–2.33)	0.61	0.434
**51–100% (SRC)**	69 (6.66)	0.14 (0.02–0.97)	3.95	**0.047**	0.14 (0.02–1.00)	3.75	**0.040**
**Surgical type**							
**Subtotal**	936 (90.35)	1.00					
**Total**	100 (9.65)	1.16 (0.60–2.24)	0.2	0.653			
**pT stage**							
**T1**	757 (73.07)	1.00			1.00		
**T2**	279 (26.93)	3.54 (2.39–5.23)	40.11	**<0.001**	3.38 (2.28–5)	36.94	**<0.001**
**pN stage**							
**N0**	976 (94.21)	1.00					
**N1**	60 (5.79)	1.86 (0.97–3.57)	3.46	0.063			
**Lymphovascular invasion**							
**Yes**	57 (5.5)	1.50 (0.70–3.23)	1.07	0.302			
**No**	979 (94.5)	1.00					
**Perineural invasion**							
**Yes**	11 (1.06)	1.19 (0.17–8.54)	0.03	0.862			
**No**	1,025 (98.94)	1.00					

*GC, gastric carcinoma; AC, adenocarcinoma; SRC, signet ring cell; CI, confidence interval; pT, pathological tumor; pN, pathological node; pTNM, pathological tumor–node–metastasis*.

*All p < 0.05 are in bold print*.

### Univariate and Multivariate Survival Analysis of Overall Survival in Patients With Stage II/III Gastric Cancer

To investigate whether the SRC components show different significance in advanced GC patients, we subsequently evaluated clinicopathological factors associated with OS in GC patients in stage II/III. In univariate analysis, age >60 (*p* = 0.000), whole stomach tumor (*p* = 0.004), SRC proportion 1–50% (*p* = 0.021), SRC proportion >50% (*p* = 0.000), total or combined organ gastrectomy (*p* = 0.000, 0.017, respectively), Borrmann's type IV (*p* = 0.000), pT stage (*p* = 0.029, 0.016, 0.002, respectively), pN stage (*p* = 0.014, 0.000, 0.000, respectively), pTNM stage (*p* = 0.000), lymphovascular invasion (*p* = 0.000), and perineural invasion (*p* = 0.015) were prognostic factors for OS (Table 2). In multivariate survival analysis, age >60 (*p* < 0.001), SRC proportion 1%−50% (*p* = 0.023), SRC proportion >50% (*p* = 0.000), total or combined organ gastrectomy (*p* < 0.001, 0.067, respectively), Borrmann's type II (*p* = 0.019), pT stage (*p* = 0.035, 0.020, 0.002, respectively), pN stage (*p* = 0.016, 0.000, respectively), and lymphovascular invasion (*p* < 0.001) were independent prognostic factors for OS (Table 3).

**Table 3:** Univariate and multivariate Cox regression analysis for OS of patients with stage II/III GC.

**Variables**	**No. of patients** **(*n* = 3,103)**	**Univariate analysis**			**Multivariate analysis**		
		**Hazard ratio** **(95% CI)**	**χ^2^**	***p-*value**	**Hazard ratio** **(95% CI)**	**χ^2^**	***p-*value**
**Gender**							
**Male**	2,416 (77.86)	1.00					
**Female**	687 (22.14)	0.95 (0.81–1.11)	0.45	0.503			
**Age (years)**							
**≤60**	1,396 (44.99)	1.00			1.00		
**>60**	1,707 (55.01)	1.31 (1.15–1.50)	15.66	**<0.001**	1.41 (1.23–1.62)	23.84	**<0.001**
**Tumor location**							
**Upper third**	1,480 (47.7)	1.00			1.00		
**Middle third**	605 (19.5)	1.14 (0.96–1.36)	2.12	0.145	0.89 (0.73–1.09)	1.21	0.271
**Lower third**	946 (30.49)	1.05 (0.90–1.23)	0.44	0.507	1.1 (0.93–1.29)	1.30	0.255
**Whole stomach**	72 (2.32)	1.71 (1.18–2.48)	8.07	**0.004**	0.9 (0.6–1.35)	0.25	0.617
**SRC proportion**							
**0% (pure AC)**	2,619 (84.4)	1.00			1.00		
**1–50% (mixed-SRC)**	299 (9.64)	1.29 (1.04–1.61)	5.29	**0.021**	1.30 (1.04–1.62)	5.17	**0.023**
**51–100% (SRC)**	185 (5.96)	1.62 (1.27–2.05)	15.68	**<0.001**	1.60 (1.26–2.03)	14.56	**<0.001**
**Surgical type**							
**Subtotal**	2,351 (75.77)	1.00			1.00		
**Total**	741 (23.88)	1.71 (1.48–1.97)	52.93	**<0.001**	1.49 (1.25–1.77)	19.88	**<0.001**
**Combined organs**	11 (0.35)	2.66 (1.19–5.94)	5.71	**0.017**	2.16 (0.95–4.91)	3.37	0.067
**Borrmann's type**							
**I**	349 (11.42)	1.00			1.00		
**II**	438 (14.33)	0.82 (0.62–1.09)	1.84	0.175	0.71 (0.53–0.95)	5.51	**0.019**
**III**	1,790 (58.55)	1.05 (0.85–1.29)	0.17	0.680	0.89 (0.72–1.1)	1.17	0.279
**IV**	480 (15.7)	1.67 (1.31–2.12)	17.28	**<0.001**	1.15 (0.9–1.48)	1.22	0.270
**pT stage**							
**T1**	48 (1.55)	1.00			1.00		
**T2**	229 (7.38)	4.82 (1.17–19.88)	4.75	**0.029**	4.58 (1.11–18.87)	4.43	**0.035**
**T3**	833 (26.84)	5.52 (1.37–22.19)	5.8	**0.016**	5.20 (1.29–20.91)	5.40	**0.020**
**T4**	1,993 (64.23)	9.21 (2.30–36.80)	9.87	**0.002**	8.73 (2.18–34.90)	9.39	**0.002**
**pN stage**							
**N0**	682 (21.98)	1.00			1.00		
**N1**	623 (20.08)	1.37 (1.07–1.76)	6.08	**0.014**	1.19 (0.85–1.67)	1.05	0.306
**N2**	750 (24.17)	2.06 (1.64–2.58)	38.61	**<0.001**	1.59 (1.09–2.31)	5.79	**0.016**
**N3**	1,048 (33.77)	3.14 (2.55–3.88)	115.07	**<0.001**	1.97 (1.35–2.86)	12.48	**<0.001**
**pTNM stage**							
**II**	1,034 (33.32)	1.00			1.00		
**III**	2,069 (66.68)	2.72 (2.29–3.22)	130.21	**<0.001**	1.41 (1.00–1.98)	3.83	0.050
**Lymphovascular invasion**							
**Yes**	574 (18.5)	1.88 (1.61–2.19)	65.1	**<0.001**	1.45 (1.23–1.71)	20.10	**<0.001**
**No**	2,529 (81.5)	1.00			1.00		
**Perineural invasion**							
**Yes**	158 (5.09)	1.41 (1.07–1.87)	5.89	**0.015**	1.19 (0.89–1.59)	1.33	0.249
**No**	2,945 (94.91)	1.00			1.00		
**Adjuvant therapy**							
**Yes**	2,093 (67.45)	0.82 (0.62–1.09)	1.84	0.175			
**No**	1,010 (32.55)	1.00					

*GC, gastric carcinoma; AC, adenocarcinoma; SRC, signet ring cell; CI, confidence interval; pT, pathological tumor; pN, pathological node; pTNM, pathological tumor–node–metastasis*.

*All p < 0.05 are in bold print*.

### Long-Term Outcomes of Different Subgroups According to the Proportion of Signet Ring Cell Components

**Figure 2:**
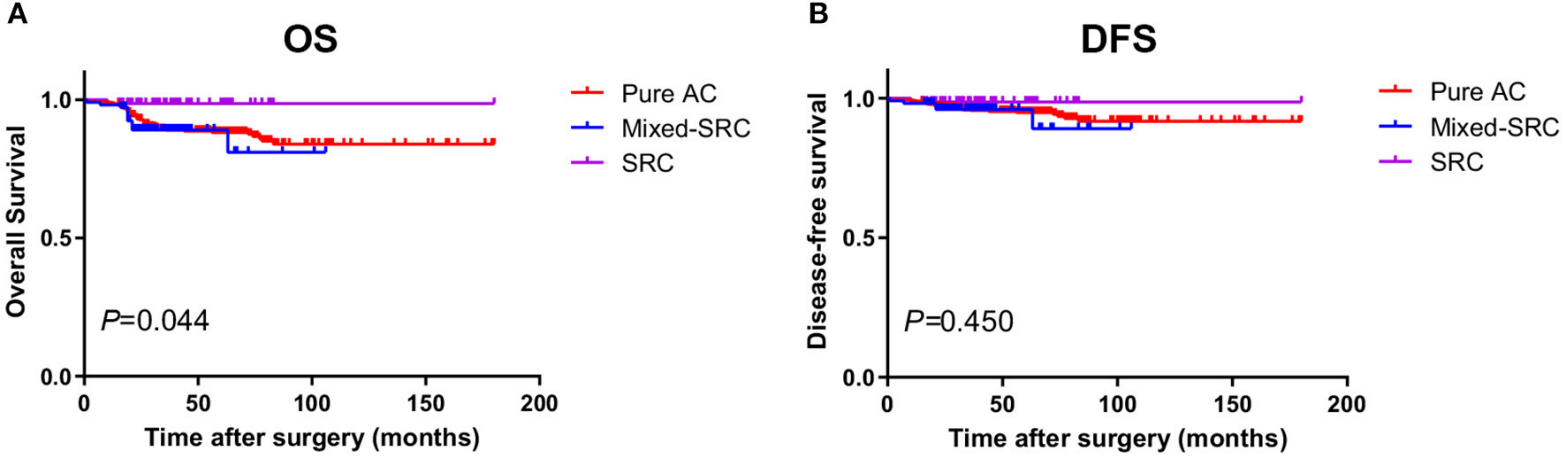
The Kaplan–Meier survival curves of overall survival (OS) **(A)** and disease-free survival (DFS) **(B)** in gastric cancer patients in stage I.

As shown in Table 4, for GC patients in stage I, the 5-year OS of patients with mixed-SRC was lower than that of patients with pure adenocarcinoma (82.95 vs. 85.15%), but the difference was not statistically significant (*p* = 0.867). However, patients with SRC had significantly higher 5-year OS than patients with pure adenocarcinoma (97.73 vs. 85.15%, *p* = 0.021). There was no statistical significance between the 5-year DFS of patients with mixed-SRC/SRC and pure adenocarcinoma (*p* = 0.824, 0.204, respectively). The trends in the Kaplan–Meier survival curves of OS and DFS are shown in Figure 2.

**Table 4:** Comparison of the 5-year OS/DFS rate according to different subgroups.

	**5-year OS rate (%)**	**χ^2^**	***p-*value**	**5-year DFS rate (%)**	**χ^2^**	***p-*value**
**Stage I**						
**Pure AC**	85.15			93.54		
**Mixed-SRC**	82.95	0.03	0.867	93.60	0.05	0.824
**SRC**	97.73	5.32	**0.021**	97.73	1.61	0.204
**Stage II/III**						
**Pure AC**	66.33			67.42		
**Mixed-SRC**	59.43	0.63	0.427	62.29	0.08	0.775
**SRC**	51.61	16.80	**<0.001**	52.75	16.87	**<0.001**

*AC, adenocarcinoma; SRC, signet ring cell; OS, overall survival; DFS, disease-free survival*.

*All p <0.05 are in bold print*.

**Figure 3:**
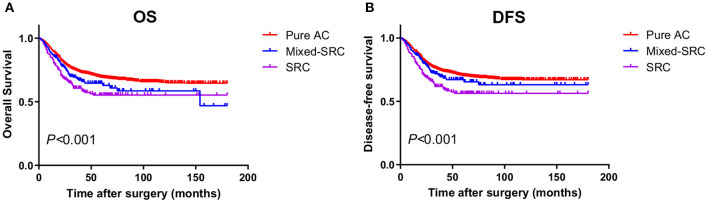
The Kaplan–Meier survival curves of OS **(A)** and DFS **(B)** in gastric cancer patients in stage II/III.

For GC patients in stage II/III, there was no statistical significance between the 5-year OS of patients with mixed-SRC and pure adenocarcinoma (59.43 vs. 66.33%, *p* = 0.427). However, patients with SRC had significantly lower 5-year OS than patients with pure adenocarcinoma (51.61 vs. 66.33%, *p* < 0.001). There was no statistical significance between the 5-year DFS of patients with mixed-SRC and pure adenocarcinoma (62.29 vs. 67.42%, *p* = 0.775). However, the 5-year DFS of patients with SRC was significantly lower than in patients with pure adenocarcinoma (52.75 vs. 67.42%, *p* < 0.001). The trends in the Kaplan–Meier survival curves of OS and DFS are shown in Figure 3.

## Discussion

Although the WHO pathological diagnostic standards define SRC as the pathologic component of more than 50% of the whole tumor, patients of gastric adenocarcinoma mixed with SRC of <50% proportion can often be observed in the clinic, which can be defined as mixed-SRC (10). It has been reported that the SRCs constitute ~5–10% of all GC cases (2). In our study, the SRC accounted for 6.1% (254/4,139) in all GC cases. The mixed-SRC accounted for 9.8% (406/4,139), even more common than SRC. However, the clinicopathological characteristics and long-term survival of patients with mixed-SRC are still unclear.

In this study, we analyzed clinicopathological features and survival results of GC patients according to the proportion of SRC components. Patients with different proportion of SRC components had significant differences in age, gender, tumor site, and pTNM stage (6). Patients with SRC/mixed-SRC were more associated with female, younger age, higher pN stage, and more lymphovascular invasion (all *p* < 0.05) than pure adenocarcinomas. Studies have shown that SRCs are more commonly seen in young female patients, which is consistent with our results (11). It is believed that the lymph node metastasis rate of SRC is higher than that of pure adenocarcinoma (12). Our results showed that the number of patients with SRC differentiation of pN stage 2/3 was higher than that with pure adenocarcinoma (*p* < 0.001). Moreover, lymphovascular invasion is also proved to be associated with poor prognosis (13). In general, patients with SRC differentiation showed more aggressive behavior.

The clinical characteristics of gastric SRC were generally considered as poor tumor differentiation and high malignancy (1). However, recent studies implied that gastric SRC patients of different tumor stages may have different long-term outcomes. For early GC patients, many studies indicate that SRC showed favorable prognosis (6, 14, 15). For example, Kao et al. (7) have reported that the 5-year overall survival of early SRC patients was significantly higher than that of non-SRC patients (90.7 vs. 83.2%, *p* = 0.001). In this study, the 5-year OS of SRC was 97.73%, significantly higher than pure adenocarcinoma (85.15%, *p* < 0.05) and mixed-SRC (82.95%, *p* < 0.05). Interestingly, early GC patients with mixed-SRC seemed to be more aggressive than patients with SRC or pure adenocarcinoma (10). Hwang et al. (16) found that lymph node metastasis rate of mixed-type cases was higher (20.2%) than cases of pure diffuse type (9.3%) and predominantly intestinal type (12.2%) histology. In early GC, the biological behavior of mixed SRC is more aggressive, with worse prognosis than pure SRC (17). Our results suggested that the 5-year OS of mixed-SRC is lower than pure adenocarcinoma (82.95 vs. 85.15%, *p* > 0.05) and SRC (82.95 vs. 97.73%, *p* < 0.05). Multivariate analysis and stratified analysis also showed that SRC components >50% were also independent risk factors (*p* = 0.040). These results were consistent with the previous studies, implying that different proportions of SRC components may indicate completely opposite survival outcomes. There is no clear reason to explain this phenomenon. Some researchers speculated that the driver mutations controlling the metastatic potential of SRC can occur late in the course of disease (6).

It has been proven that the prognosis of SRC is worse than pure adenocarcinoma in advanced GC patients. Due to its highly malignant traits, our results showed that the SRC had a greater impact on the prognosis of patients with stage II/III, even if the SRC proportion is below 50% (mixed-SRC). That means even a small proportion of SRC components also has a significant impact for prognosis in advanced GC patients. The results of this study showed that the adenocarcinomas with SRC differentiation had lower 5-year overall survival rate than pure adenocarcinoma in GC patients in stage II/III [51.61% (SRC)/59.43% (mixed-SRC) vs. 66.33% (pure AC), *p* < 0.001]. The results showed that for patients with advanced GC, the proportion of SRC components was closely associated with prognosis. The results of this study suggest that proportion of SRC components is also an independent risk factor in advanced GC patients. Therefore, the SRC components has a great influence on the prognosis of advanced GC patients because of its high malignant trait (18). Therefore, GC harboring the SRC components should be differentiated from conventional adenocarcinomas (19, 20).

In recent years, endoscopic resection (ER) has become an important option for patients with early gastric cancer (EGC). According to the latest 2018 Japanese Gastric Cancer Treatment Guidelines (5th edition), the main decisive factors of ER criteria are histological types, depth of invasion (pT stage), ulcerative findings, and tumor diameter (21). Well or moderately differentiated EGC usually means low-risk lymph node metastasis (LNM) and curative resection. Patients with SRC were thought to be not suitable for ER, but recent studies have shown the low risk of lymph node metastasis and favorable prognosis of SRC, indicating that ER can be treated as a curative resection for early SRC patients. Furthermore, according to the endoscopic resection curability (eCura) criteria (22), EGC patients who met the absolute or expanded criteria for ER, receiving en-bloc ER with negative horizontal/vertical margin and had no lymphovascular infiltration, should be regarded as suitable candidates for endoscopic treatment (23). However, the feasibility of ER in patients with histological mixed-SRC type is still unclear. Horiuchi et al. (24) believed that mixed poorly differentiated adenocarcinoma in EGC predicts endoscopic noncurative resection. Our results suggested that there was no statistical significance between patients with mixed-SRC and with pure adenocarcinoma (82.95 vs. 85.15%, *p* > 0.05). This may indicate the suitability of mixed-SRC for ER in EGC patients (25).

For GC patients in stage II/III, radical resection is essential for the treatment of GC, but even if tumors are completely removed, there may be recurrence or distant metastasis of the tumors in the following years (26). Studies have reported the benefit of adjuvant chemotherapy based on fluorouracil regimens in GC patients (27). In recent years, various large-scale phase III clinical trials have confirmed the role of adjuvant treatment for GC. However, the benefit of clinical trials based on the S-1 and XELOX regimens was only seen in the Asiatic population (28). In our study, 67.45% of the patients in stage II/III received adjuvant chemotherapy after surgery. However, those who received postoperative chemotherapy did not show better survival than others (HR 0.82, 95% CI: 0.62–1.09, *p* = 0.175). There are some data in the literature demonstrating that GC patients with SRC components might not benefit from adjuvant chemotherapy (3). The absence of benefit of adjuvant chemotherapy for advanced GC patients in our study might be due to the inclusion of these cases. Recent research data show that gastric SRCs are significantly more sensitive to mitomycin C, doxorubicin, and docetaxel, but not sensitive to fluorouracil and cisplatin (29). The future research direction of adjuvant treatment of GC should gradually be individualized (30).

In conclusion, this study was designed to retrospectively analyze the clinicopathological features and prognosis of different proportions of SRC components in GC patients. The results showed that the presence of SRC components was related to favorable prognosis in GC patients in stage I, but lower 5-year OS/DFS in GC patients in stage II/III, independent of other clinicopathological features. Therefore, GC patients with SRC components should draw clinicians' attention.

## Data Availability Statement

The raw data supporting the conclusions of this article will be made available by the authors, without undue reservation.

## Ethics Statement

The studies involving human participants were reviewed and approved by the Ethics Committee of Qilu Hospital of Shandong University. The patients/participants provided their written informed consent to participate in this study.

## Author Contributions

GS designed this study. XD and GS contributed in the drafting of the manuscript. HQ, QH, and GS critically revised the manuscript for important intellectual content. GS and ZH collected and analyzed the data. All the authors approved the final manuscript submitted. Each author participated sufficiently in the work to take public responsibility for appropriate portions of the content and agreed to be accountable for all aspects of the work in ensuring that questions related to the accuracy or integrity of any part of the work are appropriately investigated and resolved.

## Conflict of Interest

The authors declare that the research was conducted in the absence of any commercial or financial relationships that could be construed as a potential conflict of interest.
